# JAGN1 mutation with distinct clinical features; two case reports and literature review

**DOI:** 10.1186/s12887-023-04024-y

**Published:** 2023-04-29

**Authors:** Mahsa Hojabri, Yeganeh Farsi, Mahnaz Jamee, Hassan Abolhassani, Hedieh Haji Khodaverdi Khani, Abdollah Karimi, Mehrnaz Mesdaghi, Zahra Chavoshzadeh, Samin Sharafian

**Affiliations:** 1 Student Research Committee, School of Medicine, Shahid Beheshti University of Medical Sciences, Tehran, Iran; 2 Pediatric Nephrology Research Center, Research Institute for Children’s Health, Shahid Beheshti University of Medical Sciences, Tehran, Iran; 3 Immunology and Allergy Department, Mofid Children’s Hospital, Shahid Beheshti University of Medical Sciences, Tehran, Iran; 4 Department of Biosciences and Nutrition, Karolinska Institutet, Huddinge, Sweden; 5 Department of Immunology, Faculty of Medical Sciences, Shahed University, Tehran, Iran; 6 Pediatric Infections Research Center, Research Institute for Children Health, Shahid Beheshti University of Medical Sciences, Tehran, Iran

**Keywords:** Jagunal homolog 1, Severe congenital neutropenia, JAGN1, Inborn error of immunity

## Abstract

Jagunal homolog 1 (JAGN1) has been recognized as an essential protein in neutrophil function. The mutated JAGN1 is responsible for immunodeficiency related to innate and humoral defense mechanisms. This deficiency impairs neutrophil development and function, leading to recurrent infections and facial dysmorphism as phenotypic consequences of severe congenital neutropenia (SCN). We report two siblings having the reported *JAGN1* mutation with different clinical manifestations. Recurrent abscess formation unresponsive to antibiotic therapy, a history of delayed umbilical separation, frequent bacterial or fungal infection, dysmorphic face, failure to thrive, and other coexisting organ abnormalities should prompt physicians to syndromic immunodeficiencies involving neutrophils. Genetic investigations to elucidate the responsible mutation is critical as clinical management varies. Once the diagnosis is confirmed, a multi-disciplinary team should perform further workups to investigate other coexisting malformations and neurodevelopmental evaluation.

**Supplementary Information:**

The online version contains supplementary material available at 10.1186/s12887-023-04024-y.

## Introduction

Neutrophils are one of the most important cells of innate immunity associated with host defense against bacteria and fungi. Severe congenital neutropenia (SCN), a rare group of primary immunodeficiency leading to recurrent infections, highlights the critical role of neutrophils [1–3]. Numerous genes with several encoded proteins widespread in different cellular organelles have been reported to be involved in SCN. The endoplasmic reticulum (ER) is one of the most critical infra-cellular structures with a wide variety of functions and mutations in coding genes neutrophil elastase (*ELANE*), Glucose-6-phosphatase catalytic 3 (*G6PC3*), and Jagunal homolog 1 (*JAGN1*) are associated with SCN (Table 1) [4–6].

**Table 1:** The severe congenital neutropenia subtypes and their phenotype [18]

Inheritance	Responsible gene	Condition	Bone Marrow Aspiration result	Other notable features
Autosomal Recessive	*CSF3R*	SCN7	R308C mutant protein was retained in the endoplasmic reticulum and not expressed on the plasma membrane.	-
	*JAGN1*	SCN6	The maturational arrest of granulocytes at the promyelocyte/myelocyte stage.	Failure to thrive, Skin abscess
	*VPS45*	SCN5	Mutant fibroblasts and bone marrow cells showed increased apoptosis compared to controls.	Hepatosplenomegaly, Extramedullary hematopoiesis, Psychomotor retardation, Developmental delay, Hypergammaglobulinemia
	*G6PC3*	SCN4	-Decreased mature neutrophils. The predominance of atypical mononuclear megakaryocytes, myeloid hyperplasia, and vacuolization of the myeloid precursors.	Microcephaly, Hearing loss, Developmental delay, Cardiovascular defects, Hepatosplenomegaly, Genitourinary abnormalities, Skeletal dysplasia, Endocrine disorders
	*HAX1*	SCN3	-	Psychomotor retardation, Seizure, Increased risk of leukemia
Autosomal Dominant	*GFI1*	SCN2	-	Pyogenic abscess, Cyclic neutropenia in some cases
	*ELANE*	SCN1	-	-
	*SRP54*	SCN8	-Decreased levels of SRP54 mRNA -Variably reduced GTPase activity of the mutant proteins compared to controls.	Growth and developmental retardation, Autism spectrum disorder, Hypotonia, Pancreatic dysfunction, Gingivostomatitis
X-linked recessive	*WAS*	SCNX	-	Recurrent Severe infection, Abnormal T cell function, and number, Normal/ decreased IgA levels

The homozygous mutation in the *JAGN1* gene impairs the protein trafficking between the ER and the Golgi apparatus, which is critical for neutrophil homeostasis [2, 6, 7]. Recent evidence also provides the role of *JAGN1* mutation in the calpain-dependent apoptosis of neutrophils [7]. Abnormalities in N-glycosylation of proteins such as IgG have been reported so far [8, 9]. Most mutations are missense; however, the nonsense mutation is also reported [4, 5].

Patients with SCN have a history of recurrent respiratory infections, otitis, sinusitis, skin abscess, and oral thrush [4–6]. Multiple organs are also involved in this disease in which the patients have facial, cardiac, renal, urogenital, musculoskeletal, and neurodevelopmental abnormalities in the physical examination [10].

The *JAGN1*-associated neutropenia is a rare subtype of SCN that classically presents with facial dysmorphism accompanied by multisystem involvement [6, 9]. Ocular involvement, such as monocular convergent strabismus, is also seen in patients with *JAGN1* mutation [4].

Initial treatment for neutropenic patients involves administering granulocyte colony-stimulating factor (G-CSF). However, patients with *JAGN1* mutation show poor response to G-CSF therapy, and hematopoietic stem cell therapy (H-SCT) is the ultimate treatment choice [5].

Herein, we report two siblings with *JAGN1* mutation with their clinical course and distinct features and review the current literature about *JAGN1* mutation cases.

### Case presentations

Patient 1 was the third child of non-consanguineous parents with a history of delayed cord separation and omphalitis, recurrent episodes of cutaneous infections, otitis, gastrointestinal infections, and pneumonia within the first three months of life.

At five months of age, he was suspected of having an immunodeficiency due to repeated episodes of infections and hospitalization. The physical examination revealed wide fontanelles, allergic shiners, the absence of tonsils, postnasal drip, and hypotonia. The growth curve showed a failure to thrive (FTT) (weight at five months of age was 4400 gr while his birth weight was 3250 gr.). Paraclinical examinations revealed moderate neutropenia (absolute neutrophil count (ANC) = 840 cells/mm^3^). The urine analysis and culture exhibited candida infection, and the stool exam (S/E) was normal. FTT-associated workups were normal, including the sweet chloride test and metabolic screening panel. Detailed laboratory testing is demonstrated in Table S1. Bone marrow aspiration revealed myeloid suppression with cellular hypoplasia with no malignancy or aplastic anemia. Due to persistent neutropenia and clinical suspicion of syndromic disorders, we thoroughly investigated probable accompanying cardiac anomalies, genitourinary disorders, and musculoskeletal deformities and found no notable point.

When he was eight months old, widespread erythematous tender pustular eruptions on his head and back resulted in hospitalization, mild neutropenia (ANC = 1320 cells/mm^3^), anemia (hemoglobin = 8.6 g/dL), and hypogammaglobulinemia (IgG = 112 mg/dL, IgA = 10 mg/dL, IgM = not detected, IgE = 26 IU/ml) were the primary laboratory results (Table S1). Antibiotic therapy with ceftriaxone (50 mg/kg), irradiated packed red blood cell (RBC) transfusion, and intravenous immunoglobulin (IVIG, 0.4 g/kg every month) was administrated for the patient. Furthermore, we considered long-term prophylactic antibiotic therapy following the primary diagnosis of X-linked hypogammaglobulinemia. Now, the patient is a 9-year-old boy with a normal development and growth course, with no signs or symptoms of epilepsy or other neurological disorders.

A 4-year-old girl, the younger sibling of patient 1, presented with a history of multiple hospitalizations due to pneumonia and lymphadenitis drainage. She was hospitalized at two and four months of age due to pneumonia. Recurrent episodes of lymphadenopathies unresponsive to antibiotic therapy, which were all surgically drained, were one of the most considerable aspects of her history.

She was admitted to our hospital for drainage of a persistent neck abscess at 13 months old. After an episode of upper respiratory tract infection and cervical lymphadenopathy, a neck abscess formed that was unresponsive to oral antibiotic therapy. *Staphylococcus aureus* was detected in the abscess drainage culture. Based on the antibiogram test, we used vancomycin (10 mg/kg TDS), clindamycin (10 mg/kg QID), and ceftazidime (50 mg/kg TDS) to treat the residual collection after surgery.

Seven months later, another hospitalization occurred due to a unilateral swelling in the internal corner of the left eye. The computed tomography (CT) scan showed pre-orbital swelling within the left lacrimal sac. The lesion (dacryocystitis) was inflammatory and was surgically removed. Gram-negative bacillus was grown in the culture, which was treated with clindamycin (10 mg/kg QID), topical zinc oxide ointment (TDS), and cefotaxime (50 mg/kg/day).

Later on, when she was 21 months old, she was re-hospitalized due to a left axillary mass. Physical examination revealed FTT (she weighed 10 kg in 21 months while she was 3500 gr at birth.), left axillary fluctuated mass with swelling and erythema of the ipsilateral fingers. The intravenous administration treated the cellulitis of meropenem (10 mg/kg TDS) and vancomycin (10 mg/kg QID). No mycobacterium or fungi was detected in the culture of the abscess aspiration. During hospitalization, we managed otitis media with purulent secretion with ciprofloxacin otic drop (3 gutt, TDS) and dexamethasone ear drop (3 gutt, TDS).

Recurrent abscess formation, episodes of pneumonia and otitis, and a history of immunodeficiency in the sibling led us to the patient’s combined immune deficiency (CID). Immunologic workups revealed abnormal LTT tests with decreased BCG, PHA, and candida response. Anti-tetanus IgG and anti-diphtheria were 0.44 and 0.06, respectively (< 0.1 primary immunization). Transient hypogammaglobulinemia in infancy was also notable.

Whole Exome Sequencing revealed the homozygous missense variant of c.59G > A; *p*.R20Q in exon 1 of the *JAGN1* gene (8.6e-06 minor allele frequency based on gnomAD, Combined Annotation Dependent Depletion of 34, with the Mutation Significance Cutoff of 23) for both patients, confirmed by Sanger sequencing (Table 2). This variant is a known pathogenic variant reported in a previous SCN patient from the same geographical region, indicating a founder effect in the Iranian cohort of SCN with *JAGN1* deficiency similar to international cohorts. The majority of patients in the world have been reported with specific mutations affecting mainly the first cytoplasmic domain of the protein, particularly p. H44Y and p.M1I identified in Algerian and Turkish cohorts, respectively.

**Table 2:** Summary of clinical presentation and course of previously reported patients with JAGN1 deficiency

Patient no. (ref)	Sex	Age	Consanguinity of parents	Clinical presentation	Physical features	Age at diagnosis	Genetic mutation	Special note	Hospitalizations	Final state
1 (6)	M	10	Yes	-GA: 37 w -BW 1900 -Sepsis -Recurrent infections: pneumonia, otitis media, sinusitis, ulcers, and abscesses on lower extremities. -Severe neutropenia after two episodes of hospitalization	-Triangular face and extrovert ears -allergic rhinoconjunctivitis with sensitization against house dust mites	Four years	Homozygous missense mutation in exon 2 of *JAGN1* gene (c 130 c > T, p. His44 Tyr)	-Nl Growth, -Nl development -No other congenital anomalies -Nl serum Ig and Lymph subtypes -Positive skin prick test against house dust mites	-Day 4: Sepsis -month 6: Bilateral abscesses on lower extremities unresponsive to Ab -3 years: Severe pneumonia and cavernous lesions on CT. antifungal + anti-TB empirically for six months.	Alive, Waiting for HSCT -High doses of G-CSF (10 µg /kg). -Hospitalization 2–3 times every year due to pneumonia. -Higher doses of G-CSF during infections.
2 (4, 6)	F	7	Yes	-Upper respiratory tract infections -Skin Abscesses	-Bilateral hip dysplasia -Extramedullary hematopoiesis with thickening of skull bones		Homozygous mutation in of *JAGN1* gene (c 130 c > T, p. His44 Tyr)			Alive and well
3 (5)	M	2	No	-Congenital neutropenia - recurrent episodes of respiratory infections, pneumonia, otitis, oral and genital candidiasis, and recurrent skin abscesses	-Dysmorphic face -Convergent monocular strabismus (syndrome of Stilling-Turk- Duane type 1) -Moderate growth (height 15th weight 40th centiles) -2/6 systolic murmur -Inguinal multiple bilateral abscesses -Extrahem-autopoietic features	Three years	Homozygous missense mutation in exon 1 of *JAGN1* gene: c.G63T (p.Glu21Asp)	-Mild neurodevelopmental delay -Aortic subvalvular membrane with mild AI -Thrombotic predisposition (MTHFR mutation with S protein reduction) -Pulmonary bronchiectasis	-2 yrs: wound swab was positive for multiresistant *Pseudomonas aeruginosa* and *Klebsiella pneumonia* -2y + 1 m: *E. Coli* & *Enterococcus* MDR -2y + 2 m: multiple episodes of Infection: genital abscesses by *P. aeruginosa*, gingivostomatitis, candidiasis, perianal cellulitis, pneumonia complicated with cavitary lesion *Morganella morganii*.	?
4 (4, 8)	M	18	Yes	-Gluteal abscess -Cervical LAP -Multiple episodes of infections: pneumonia, diarrhea, otitis, and gingivitis with no response to the therapy	-Dysmorphic face - failure to thrive -Hepatomegaly -Hypospadias -Left undescended testis -Mental retardation -Bronchiectasis -Amelogenesis imperfecta	Nine months	missense mutation in exon 2 of *JAGN1* (c.130 C > T, p. His 44Tyr)	-Hypothyroidism - High antithyroglobulin antibody -Low HDL cholesterol		Alive, Free of infection
5 (8)	F	13	Yes	-Recurrent skin abscesses -Otitis -Pneumonia	-Triangular face -Amelogenesis imperfecta -Gingival hypertrophy -Short stature -Learning disability -Hypogammaglobulinemia	Four months	missense mutation in exon 2 of *JAGN1* (c.130 C > T, p. His 44Tyr)	-Hypothyroidism		Alive, Free of infection
6 (1)	M	4	Yes	-Umbilical infection during the neonatal period -Frequent episodes of bronchitis and pneumonia		Two years				Alive, Waiting for HSCT, resistant to G-CSF with severe infections
7 (4)	F	23	Suggested	-ENT infections -Aphtosis -Perianal cellulitis -Skin abscesses			Homozygous mutation in o *JAGN1* (c. 3G > A, p. Met1Ile)			Alive and well
8 (4)	F	17	Suggested	-ENT infections	-Short stature		Homozygous mutation in o *JAGN1* (c. 3G > A, p. Met1Ile)			Alive and well
9 (4)	M	19	Suggested	-Aphtosis -Skin abscesses -Balanitis -Pneumonitis -Lung Abscess -Osteitis -Perianal cellulitis	-Pyloric stenosis		Homozygous mutation in o *JAGN1* (c. 3G > A, p. Met1 Ile)			Alive and well
10 (4)	F	17	Suggested	-Otitis -Paraodontopathy	-Scoliosis -Dental malformations		Homozygous mutation in o *JAGN1* (c. 3G > A, p. Met1 Ile)			Alive and well
11(4)	M	5	Suggested	-ENT infections -Aphtosis -Skin Abscess -Perianal cellulitis			Homozygous mutation in o *JAGN1* (c. 3G > A, p. Met1 Ile)			Alive and well
12 (4)	F	12	Yes	-Upper respiratory tract infections -Pneumonia -Skin abscesses	-Febrile convulsion -Focal epilepsy		Homozygous mutation in o *JAGN1* (c. 59G > A, p. Arg20Glu)			Alive and well
13 (4)	F	28	Yes	-Skin Abscesses -Onycholysis			Homozygous mutation in o *JAGN1* (c. 40G > A, p. Gly14Ser)			Alive and well
14 (4)	M	13	Yes	-Aspergillosis	-Severe osteoporosis -Repeated bone fractures		Homozygous mutation in o *JAGN1* (c. 297 C > G, p. Tyr99*)			Alive with H-SCT at age nine months
15 (4)	F	5	Yes	-Skin Abscesses -Omphalitis -Pancolitis	-Lipomatosis -Pancreatic insufficiency -Bone abnormalities -Dental malformations		Homozygous mutation in o *JAGN1* (c. 485 A > G, p. Gln162Arg)			Died due to pancolitis and septicemia
16 (4)	F	16	Yes	-Upper respiratory tract infections -Pneumonia -Skin abscess	-Short stature -Amelogenesis imperfecta		Homozygous mutation in o *JAGN1* (c. 63G > T, p. Glu21Asp)	Neurodevelopmental delay		Alive and well
17 (4)	F	0	No				Homozygous mutation in o *JAGN1* (c. 485 A > G, p. Gln162Arg)			Alive, waiting for H-SCT
18 (4)	F	25	No	-Pneumonia -Bronchiectasis			Homozygous mutation in o *JAGN1* (c. 35_43del CCGACGGCA, p. Thr12_Gly14del)			Alive with H-SCT at age 20 years.

## Discussion

*JAGN1* mutation is one of the rare genetic mutations responsible for severe congenital neutropenia (SCN) [11, 12]. SCN is characterized by the neutrophil maturation pathway and neutropenia impairment, leading to recurrent infections, FTT, multiple organ abnormalities, and dysfunction [11–13]. SCN is a heterogeneous condition with numerous involved genes [12]; Table 1 summarizes the inheritance pattern of SCN subtypes, the active genes, and the main features of each situation based on OMIM classification.

The *JAGN1* mutation is associated with impaired N- glycosylation of antibodies in the endoplasmic reticulum membrane, resulting in neutrophil maturation arrest, higher susceptibility to fungal invasions, and antibody dysfunction [3, 8, 14]. Moreover, mutations in *JAGN1* can affect cell viability and apoptosis by calpain-mediated mechanisms [7].

Currently, reported patients with *JAGN1* mutations have presented recurrent infection, abscess formation, structural deformities or dysfunction in various organ- systems, including FTT, short stature, neuro-developmental delay, seizure, dysmorphic face, teeth malformation, gingival hypertrophy, cardiovascular anomalies, hepatosplenomegaly, hypospadias, undescended testis, amelogenesis imperfecta, skeletal deformities, extramedullary hematopoiesis complications and symptoms [5, 6, 9]. Table 2 summarizes and compares all the reported mutant cases of *JAGN1*.

One of our patients’ most highlighted aspects is the ocular infective involvement as dacryocystitis, which has not been reported previously. The other clinical features of our patients, like recurrent abscess formation, otitis media, pneumonia, and lymphadenopathies, are identical to other registered patients with *JAGN1* mutations [5].

It is worth mentioning that despite having the same genetic mutation in the siblings in our study, they presented different clinical manifestations. While the male sibling presented with delayed cord separation, omphalitis, recurrent infections, and FTT, his younger sister had normal cord separation with recurrent persistent lymphadenopathies. Baris et al. also reported a sibling (sister and brother) with the same *JAGN1* genotype with different clinical presentations [9]. Therefore, it may be hypothesized that some sex-dependent determining factors are involved in patient phenotypes [15, 16].

The previously reported Iranian patient with *JAGN1* mutation was a girl from consanguineous parents who presented pneumonia, upper respiratory tract infection, skin abscess, and focal epilepsy with the same genetic mutation [5]. However, the siblings in this study did not show epilepsy or other neurological symptoms.

The curative strategy for *JAGN1* mutation patients is hematopoietic stem cell transplantation. While the patients classically do not respond to G- CSF, some evidence supports the partial effectiveness of G- CSF [5, 6, 9]. Cipe et al. have reported a case of *JAGN1* mutation on clinical surveillance by G-CSF and some episodes of pneumonia annually, which is managed by increasing the dose of G-CSF and proper antibiotic therapy [6]. Promising results of genetic corrective-based treatments and targeted therapies in other types of SCN have shown a new horizon in treating these patients [17].

## Conclusion

Recurrent abscess formation unresponsive to antibiotic therapy, with a history of delayed umbilical separation, frequent bacterial or fungal infection, dysmorphic face, FTT, and other coexisting organ abnormalities should prompt physicians to syndromic immunodeficiencies involving neutropenia. Genetic investigations to elucidate the responsible mutation is critical as clinical management varies. Once the diagnosis is confirmed, a multi-disciplinary team should perform further workups to investigate other coexisting malformations and evaluate neurodevelopmental states.

## Electronic supplementary material

Below is the link to the electronic supplementary material.

Supplementary Material 1

## Data Availability

The detailed laboratory data of two patients are available as supplementary material.
