# Supplementary material for: JAGN1 mutation with distinct clinical features; two case reports and literature review
Source: BMC Pediatr. 2023 Apr 29;23:206. doi: 10.1186/s12887-023-04024-y (PMC10148515; doi:10.1186/s12887-023-04024-y)
Supplement: Supplementary file 1 — Supplementary Material 1 [file 12887_2023_4024_MOESM1_ESM.docx]

**Table S1.** Immunological Laboratory findings of both patients at different time points

| **Laboratory tests** | **Patient 1** | | | | **Patient 2** | | | **unit** | **Reference value** |
| --- | --- | --- | --- | --- | --- | --- | --- | --- | --- |
|  | **5 months** | **8 months** | **7 yrs** | **9 yrs** | **13 months** | **21 months** | **4 yrs** |  |  |
| **Hb** | 11.5 | 8.6 | - | - | 8.3 | 8.5 | - | gr/dl | 13-17 |
| **Plt** | 317 | 309 | - | - | 423 | 294 | - | 10^3/ml | 150-450 |
| **WBC** | 7000 | 11000 | - | - | 8600 | 8300 | - | /µl | 4000-11000 |
| **ANC** | 840 | 1320 | - | - | 1720 | 2075 | - | - | - |
| **ALC** | 6020 | 9350 | - | - | 6192 | 5727 | - | - | - |
| **IgG level (ELISA)** | - | 112 | - | 2468 | 554 | 5.01 | 1945 | mg/dl  (Nephelometric) | 500-1300 |
| **IgA level (ELISA)** | - | 10 | - | 416 | 84 | 0.29 | 274 | mg/dl  (Nephelometric) | 48-345 |
| **IgM level (ELISA)** | - | Not detected | - | 162 | 114 | 0.64 | 119 | mg/dl | 40-180 |
| **IgE level (ELISA)** | - | 26 | - | 2 | 35 | 38.9 | 91 | IU/ml | - |
| **Isohemagglutinin level (Anti-B)** | - | - | - | - | - | 1.2 | - | - | - |
| **CD3** | - | - | 69 | 53 | 53 | - | 50 | - | 35-78% |
| **CD4/CD8** | - | - | 1.17 | 1.35 | - | - | 1.5 | - | 1-3% |
| **CD4** | - | - | 36 | 27 | 32 | - | 30 | - | 22-62% |
| **CD8** | - | - | 29 | 20 | 21 | - | 20 | - | 12-36% |
| **CD16** | - | - | 9.8 | - | 6.25 | - | - | - | - |
| **CD56** | - | - | - | - | 4.56 | - | - | - | - |
| **CD16+56** | - | - | - | 9.21% | - | - | 4.85% | - | - |
| **CD19** | - | - | 16 | 13 | 30 | - | 22 | - | 3-14% |
| **CD20** | - | - | 17 | 12.71 | 30 | - | 22 | - | 3-15% |
| **CH50** | - | - | - | - | 94 | 104 | - | - | - |
| **NBT** | - | - | - | - | 100 | 68 | - | - | - |

Hb: Hemoglobin; Plt: Platelet; WBC: White Blood Cell; ANC: Absolute Neutrophile Count; ALC: Absolute Lymphocyte Count; IgG: Immunoglobulin G; IgA: Immunoglobulin A; IgM: Immunoglobulin M; IgE: Immunoglobulin E; NBT: Nitroblue Tetrazolium test
